# Supplementary material for: Earthquake forecasting from paleoseismic records
Source: Nat Commun. 2024 Mar 2;15:1944. doi: 10.1038/s41467-024-46258-z (PMC10908837; doi:10.1038/s41467-024-46258-z)
Supplement: Supplementary file 1 — Supplementary Information [file 41467_2024_46258_MOESM1_ESM.pdf]

Earthquake forecasting from paleoseismic records

Ting Wang[1*], Jonathan D. Griffin[2], Marco Brenna[3], David Fletcher[4], Jiaxu Zeng[5], Mark Stirling[3], Peter W. Dillingham[1,6], Jie Kang[7]

[1*]Department of Mathematics and Statistics, University of Otago, Dunedin 9016, New Zealand

[2]Community Safety Branch, Geoscience Australia, Symonston 2609, ACT, Australia

[3]Department of Geology, University of Otago, Dunedin 9016, New Zealand

[4]David Fletcher Consulting Limited, 67 Stornoway Street, Karitane 9471, New Zealand

[5]Department of Preventive and Social Medicine, Otago Medical School, University of Otago, Dunedin 9016, New Zealand

[6]Coastal People: Southern Skies Centre of Research Excellence, University of Otago, Dunedin 9016, New Zealand

[7]Beef + Lamb New Zealand Genetics, 3 Crawford Street, PO Box 5501, Dunedin 9054, New Zealand

Supplementary Figures

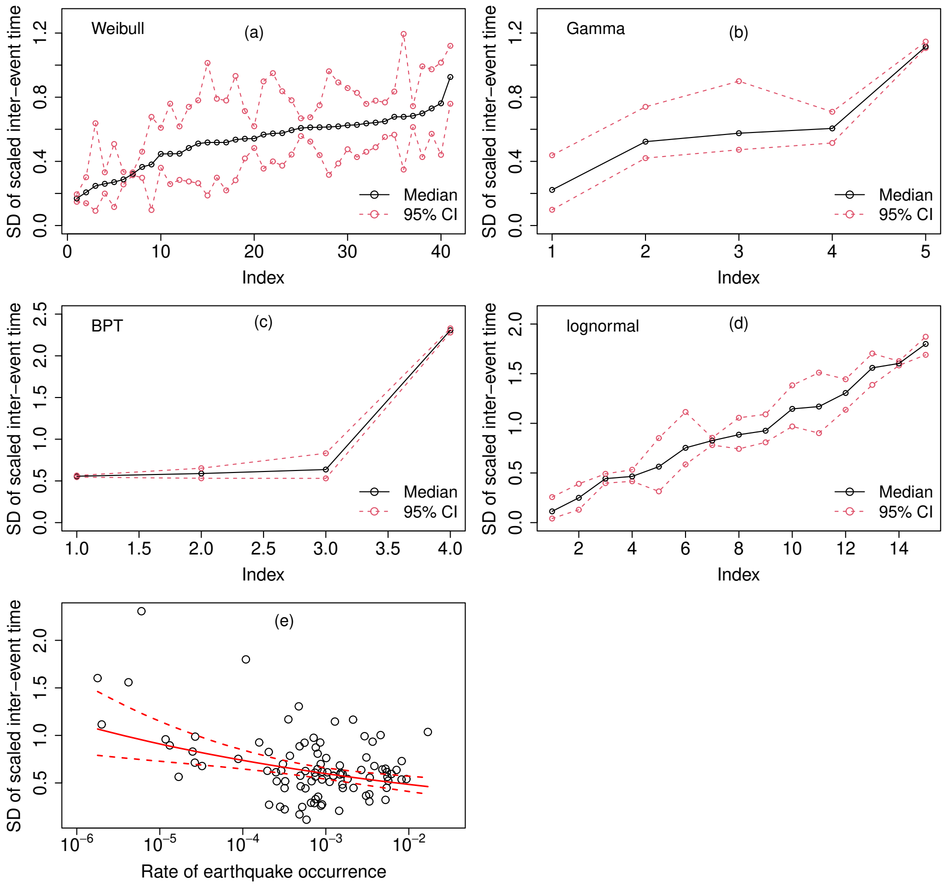

Supplementary Fig. 1. The standard deviations of the scaled inter-event times. (a)–(d): The standard deviations of the scaled (divided by the mean value) inter-event times (we calculate the scaled inter-event times for each MC sample, and report the median and 2.5% and 97.5% quantiles of the standard deviations of the scaled inter-event times calculated for the 100 MC samples) for fault segments that have the same single-best model. (a) Weibull model; (b) Gamma model; (c) Brownian passage-time (BPT) model; (d) lognormal model. We sorted the fault segments by increasing median standard deviations, so the x-axis represents index of the fault segments that have the same single-best model. This is done only for fault segments for which there is a clear single-best model (any single model having WAIC weight ≥0.95). The Poisson model doesn't have any weight over 0.7, so it is not included in the plot. (e) Standard deviations (median) of the scaled inter-event times versus rate of earthquake occurrences. The regression line and the 95% credible intervals are obtained by fitting the following model to the data, with an estimated slope b taking -0.21 (95% CI: -0.33, -0.10):

$$\hat{\lambda}_j \sim N(\lambda_j, s_j^2); \qquad \lambda_j \sim N(\mu_j, \sigma_j^2); \qquad \mu_j = a + b\ r_j$$

where $\hat{\lambda}_j$ is the posterior mean of $\lambda_j \equiv \log(SD_j)$; SD_j is the standard deviation of the scaled inter-event times for the jth fault segment; s_j^2 is the posterior standard deviation of λ_j; μ_j is the expected value of λ_j for a specified value of r_j; σ_j^2 is the process-error variance (to be estimated); a and b are regression coefficients (to be estimated); and r_j is the rate of earthquake occurrence on a log10-scale.

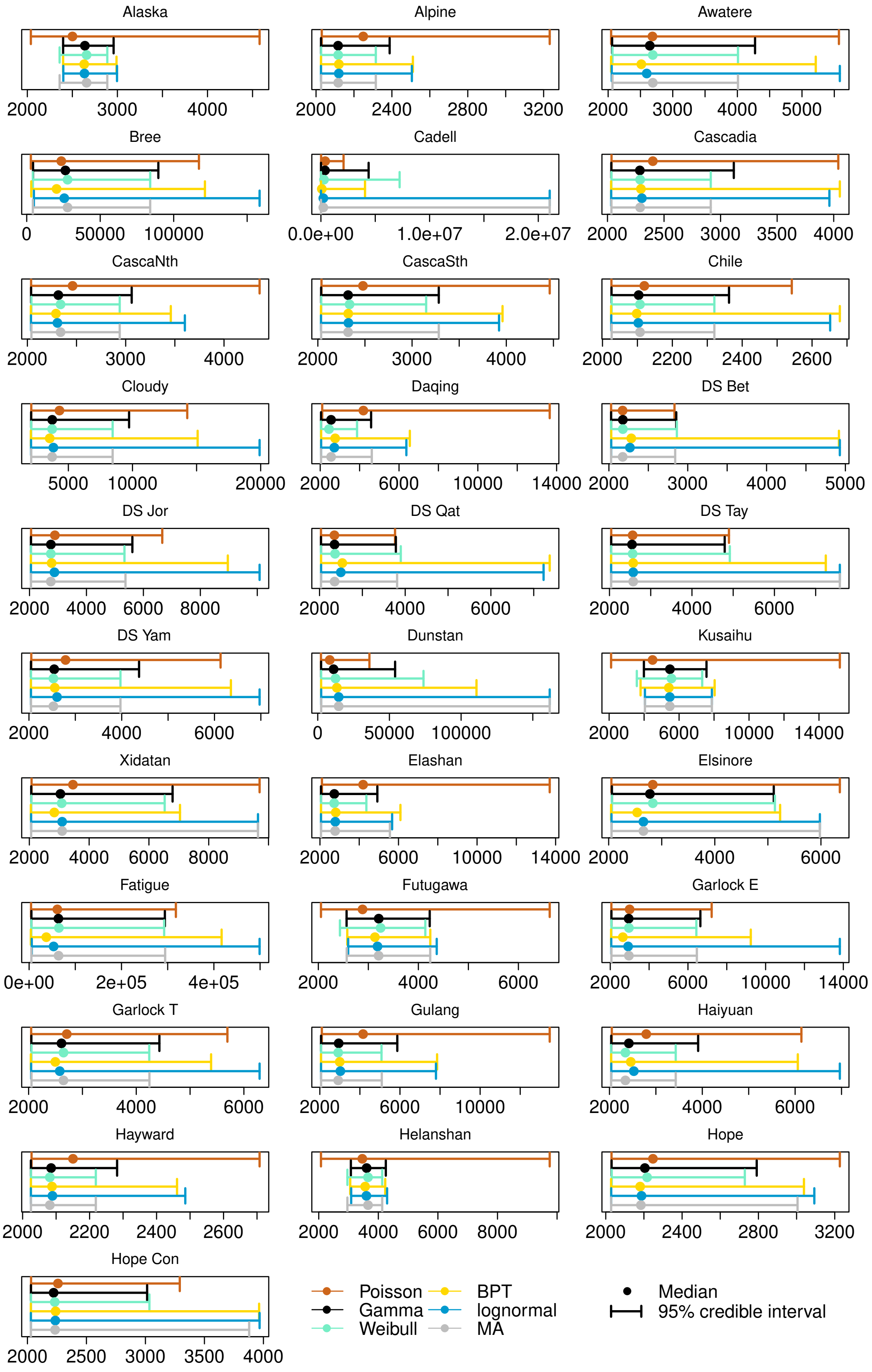

Supplementary Fig. 2. Forecast occurrence times (in years CE) of the next large earthquake from all five models and the model-averaging approach. BPT: Brownian passage-time; MA: model-averaging.

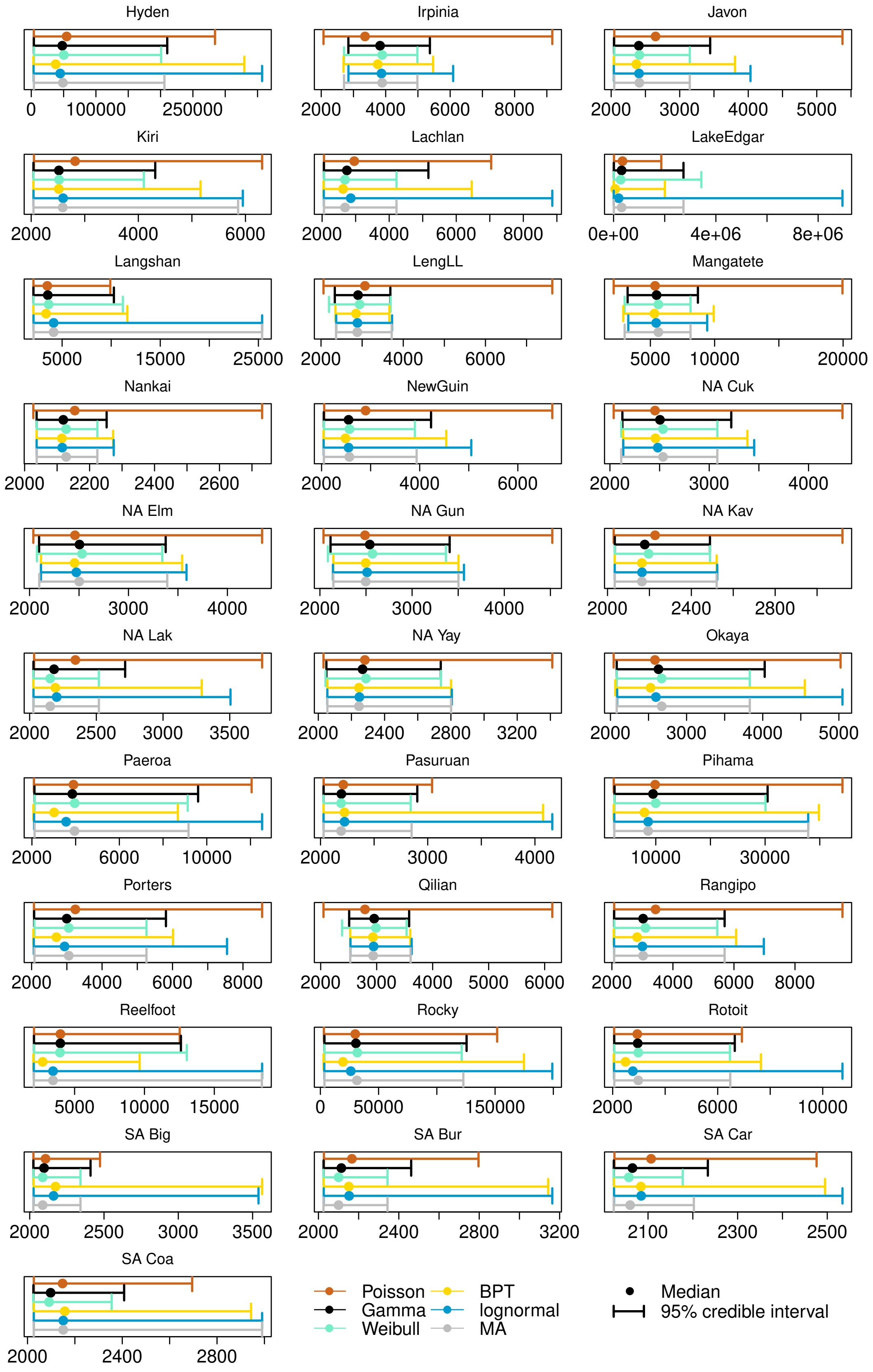

Supplementary Fig. 3. Forecast occurrence times (in years CE) of the next large earthquake from all five models and the model-averaging approach. BPT: Brownian passage-time; MA: model-averaging.

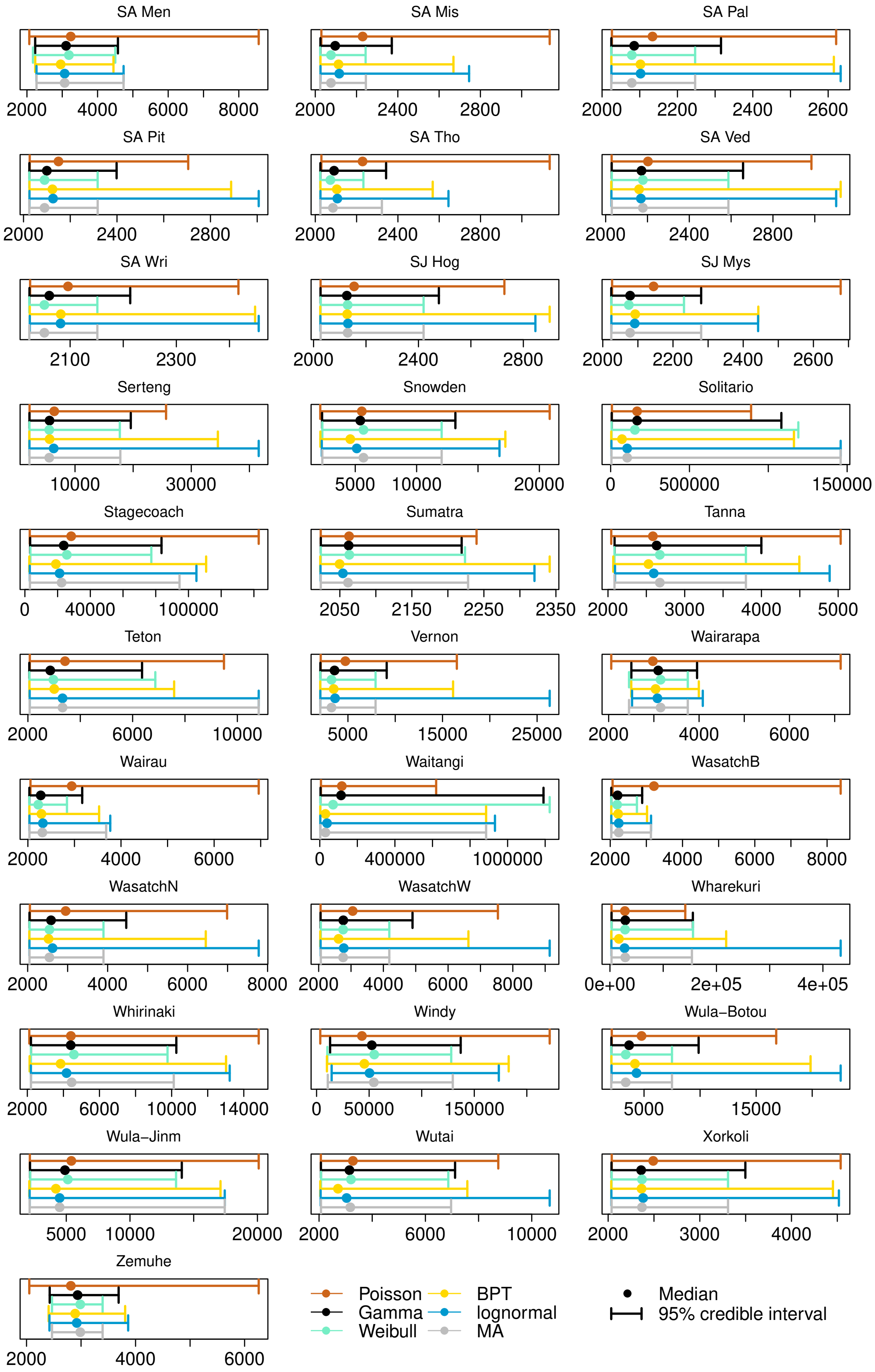

Supplementary Fig. 4. Forecast occurrence times (in years CE) of the next large earthquake from all five models and the model-averaging approach. BPT: Brownian passage-time; MA: model-averaging.

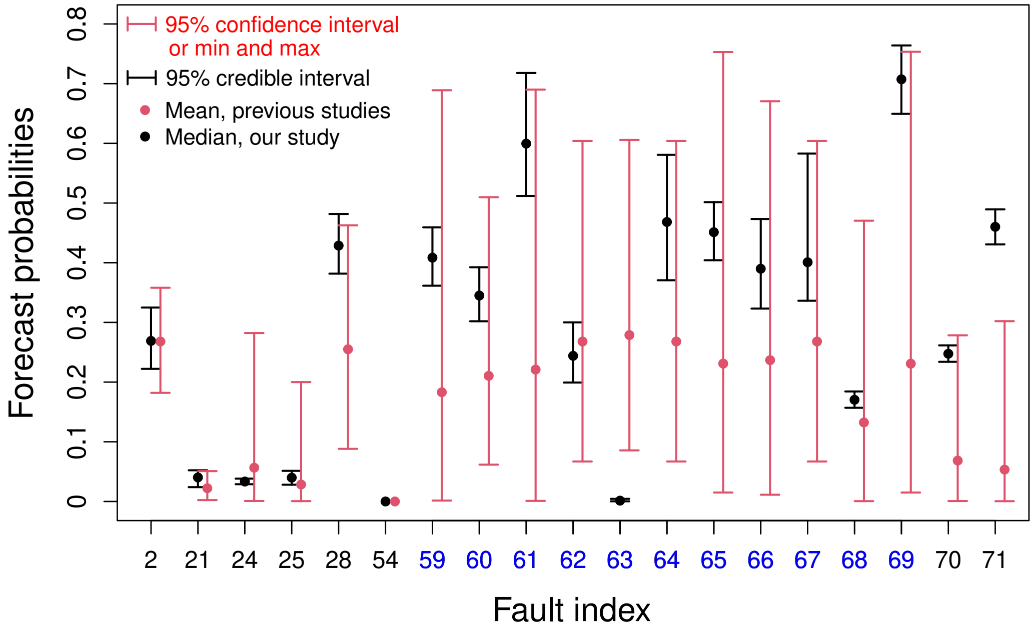

Supplementary Fig. 5. Comparison of forecast probabilities from our study with those from previous studies. The fault index in the x-axis follows the index in Table 1 in the main manuscript. San Andreas fault segments have indices from 59 to 69. The black dots are median forecast probabilities with the black bars showing 95% credible intervals. The red dots are mean forecast probabilities with the red bars showing either min and max or 95% confidence intervals. See Supplementary Table 1 for more details about the data used in this plot.

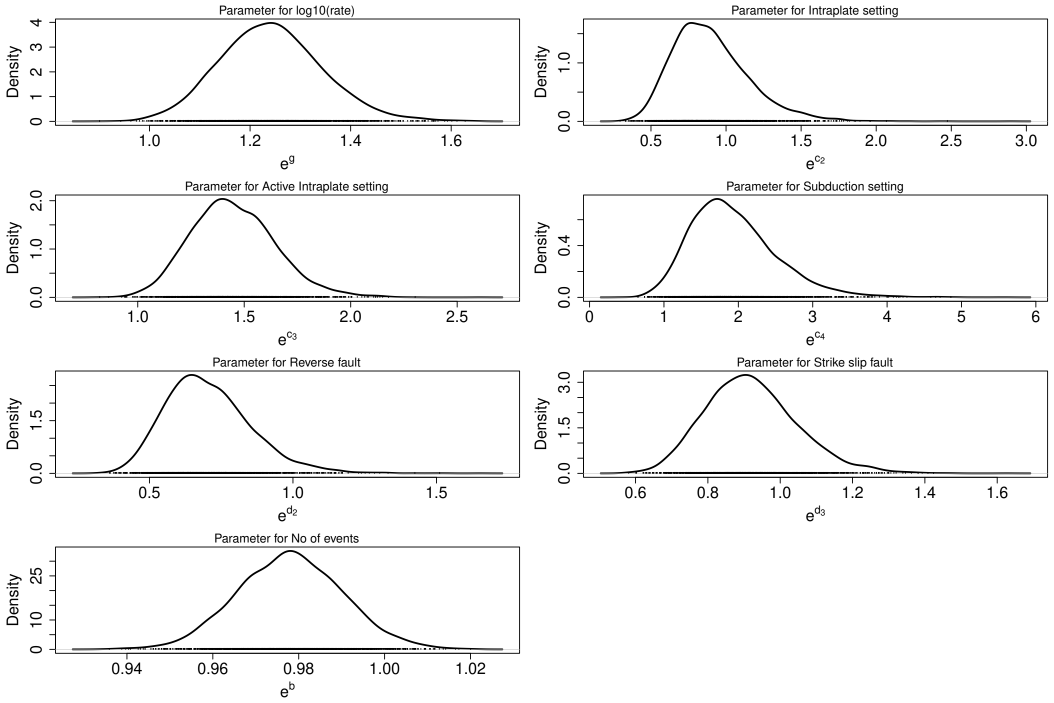

Supplementary Fig. 6. Posterior distributions of the parameters of the regression model for the relationship between the shape parameter of a Weibull renewal process and the earthquake rate, tectonic region, faulting type, and the number of earthquakes of each of the 93 fault segments. The model is

$\hat{\gamma}_j \sim N(\gamma_j, s_j^2)$

$\gamma_j \sim N(\mu_j, \sigma_j^2)$

$\mu_j = a + bN_j + c_2x_{2j} + c_3x_{3j} + c_4x_{4j} + d_2y_{2j} + d_3y_{3j} + g\ r_j$

where $\hat{\gamma}_j$ is the posterior mean of $\gamma_j \equiv \log(\alpha_j)$; α_j is the shape parameter for the jth fault segment; s_j^2 is the posterior standard deviation of γ_j; μ_j is the expected value of γ_j for specified values of the predictors; σ_j^2 is the process-error variance (to be estimated); a, b, c_2, c_3, c_4, d_2, d_3 and g are regression coefficients (to be estimated); N_j is the number of events in the catalogue for fault segment j; x_{2j}, x_{3j} and x_{4j} are dummy variables for intraplate, active intraplate and subduction tectonic settings, respectively, with at or near plate boundary as the referencing tectonic setting; y_{2j} and y_{3j} are reverse and strike slip faults, respectively, with normal fault as the referencing faulting style; and lastly r_j is the rate of earthquake occurrence on a log10-scale.

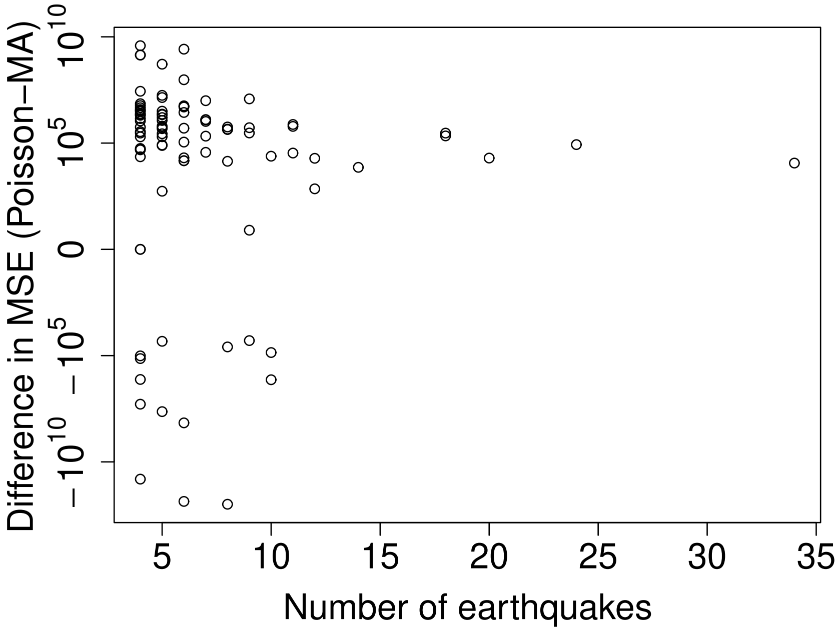

Supplementary Fig. 7. Difference between the mean squared errors of the Poisson retrospective forecasts and the model-averaging retrospective forecasts. The y axis: Difference between the mean squared errors (average squared difference between the forecast values and the true value, which is the sum of the variance and the bias squared) of the Poisson retrospective forecasts and the model-averaging retrospective forecasts. Positive values indicate that the Poisson forecasts have much larger mean squared errors. The x axis: number of earthquakes in the paleo records used in the retrospective forecasts.

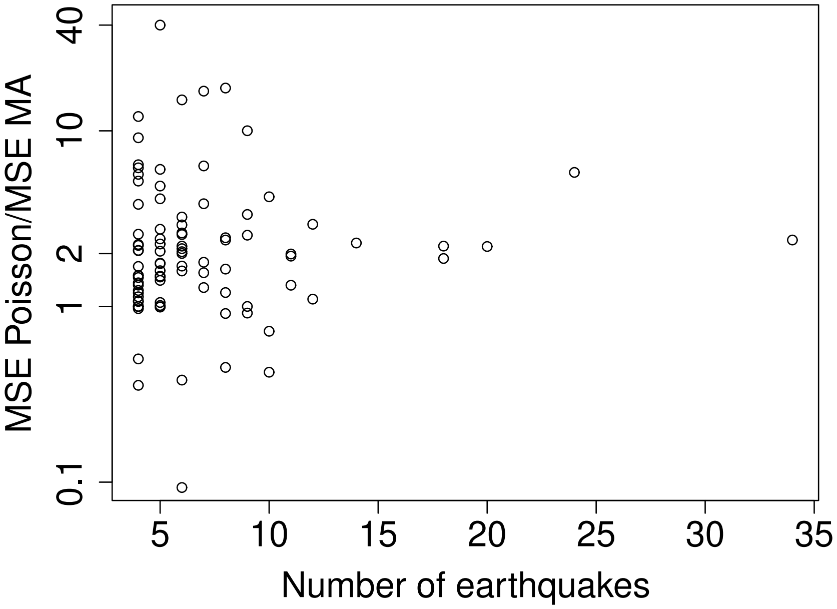

Supplementary Fig. 8. Difference between the mean squared errors of the Poisson retrospective forecasts and the model-averaging retrospective forecasts. The y axis: Relative difference between the mean squared errors (average squared difference between the forecast values and the true value, which is the sum of the variance and the bias squared) of the Poisson retrospective forecasts and the model-averaging retrospective forecasts. Values above 1 indicate that the Poisson forecasts have much larger mean squared errors. The x axis: number of earthquakes in the paleo records used in the retrospective forecasts.

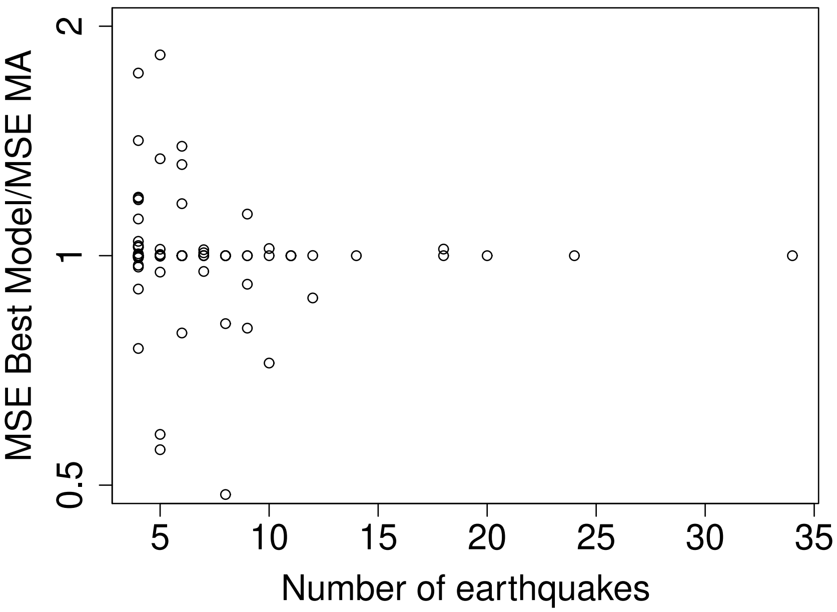

Supplementary Fig. 9. Difference between the mean squared errors of the single-best model retrospective forecasts and the model-averaging retrospective forecasts. The y axis: Relative difference between the mean squared errors (average squared difference between the forecast values and the true value, which is the sum of the variance and the bias squared) of the single-best model retrospective forecasts and the model-averaging retrospective forecasts. Values above 1 indicate that the single-best model forecasts have much larger mean squared errors. The x axis: number of earthquakes in the paleo records used in the retrospective forecasts.
