# Supplementary material for: Earthquake forecasting from paleoseismic records
Source: Nat Commun. 2024 Mar 2;15:1944. doi: 10.1038/s41467-024-46258-z (PMC10908837; doi:10.1038/s41467-024-46258-z)
Supplement: Supplementary file 3 — Description of Additional Supplementary Files [file 41467_2024_46258_MOESM3_ESM.pdf]

Description of Additional Supplementary Files

File Name: Supplementary Data 1
Description: A quantitative comparison of our probabilistic forecasts with those published in other studies.
